# Evaluation of the Wear of Ni 200 Alloy After Long-Term Carbon Capture in Molten Salts Process

**DOI:** 10.3390/ma17246302

**Published:** 2024-12-23

**Authors:** Piotr Palimąka, Stanisław Pietrzyk, Maciej Balcerzak, Krzysztof Żaba, Beata Leszczyńska-Madej, Justyna Jaskowska-Lemańska

**Affiliations:** 1 Department of Physical Chemistry and Metallurgy of Non-Ferrous Metals, Faculty of Non-Ferrous Metals, AGH University of Krakow, al. Adama Mickiewicza 30, 30-059 Cracow, Poland; pietstan@agh.edu.pl; 2 Department of Metal Working and Physical Metallurgy of Non-Ferrous Metals, Faculty of Non-Ferrous Metals, AGH University of Krakow, al. Adama Mickiewicza 30, 30-059 Cracow, Poland; balcerzak@agh.edu.pl (M.B.); krzyzaba@agh.edu.pl (K.Ż.); 3 Department of Materials Science and Engineering of Non-Ferrous Metals, Faculty of Non-Ferrous Metals, AGH University of Krakow, al. Adama Mickiewicza 30, 30-059 Cracow, Poland; bleszcz@agh.edu.pl; 4 Department of Geomechanics, Civil Engineering and Geotechnics, Faculty of Civil Engineering and Resource Management, AGH University of Krakow, al. Adama Mickiewicza 30, 30-059 Cracow, Poland; lemanska@agh.edu.pl

**Keywords:** CO_2_ capture, molten salts, CCMS process, CCS/CCU technologies, NDT methods

## Abstract

Reducing CO_2_ emissions is one of the major challenges facing the modern world. The overall goal is to limit global warming and prevent catastrophic climate change. One of the many methods for reducing carbon dioxide emissions involves capturing, utilizing, and storing it at the source. The Carbon Capture in Molten Salts (CCMS) technique is considered potentially attractive and promising, although it has so far only been tested at the laboratory scale. This study evaluates the wear of the main structural components of a prototype for CO_2_ capture in molten salts—a device designed and tested in the laboratories of AGH University of Kraków. The evaluation focused on a gas barbotage lance and a reactor chamber (made from Nickel 200 Alloy), which were in continuous, long-term (800 h) contact with molten salts CaCl_2_-CaF_2_-CaO-CaCO_3_ at temperatures of 700–940 °C in an atmosphere of N_2_-CO_2_. The research used light microscopy, SEM, X-ray, computed tomography (CT), and 3D scanning. The results indicate the greatest wear on the part of the lance submerged in the molten salts (3.9 mm/year). The most likely wear mechanism involves grain growth and intergranular corrosion. Nickel reactions with the aggressive salt environment and its components cannot be ruled out. Additionally, the applied research methods enabled the identification of material discontinuities in the reactor chamber (mainly in welded areas), pitting on its surface, and uneven wear in different zones.

## 1. Introduction

Carbon dioxide is one of the greenhouse gases responsible for the increase in Earth’s average temperature and the resulting global warming [1,2,3,4]. The primary sources of CO_2_ emissions into the atmosphere are the energy, transportation, and industrial sectors, which rely on fossil fuel combustion. In the pursuit of climate neutrality, reducing CO_2_ emissions across all areas of economic activity, as well as removing it where reduction is challenging, plays a crucial role. By reducing CO_2_ emissions to the atmosphere and shifting to zero-emission energy sources, we can prevent the future catastrophic effects of climate warming on our planet [5]. There are four ways to reduce carbon dioxide emissions [6]:

- Limiting the use of fossil fuels (improving energy conversion efficiency, reducing energy demand, and using renewable energy sources);
- Increasing the use of green hydrogen;
- Replacing fossil fuels with gaseous fuels;
- Reducing deforestation, thereby storing more CO_2_ in biomass;
- Capturing CO_2_ generated from fuel combustion in power plants and other industrial processes and storing it in suitable geological formations (CCS—Carbon Capture and Storage) and/or using it to enhance oil recovery (EOR—Enhanced Oil Recovery).

The Intergovernmental Panel on Climate Change (IPCC) consistently includes CCS technologies as one of the pathways for mitigating CO_2_ emissions, in line with the goals set by the Paris Agreement [7]. The main contribution of CCS technologies to achieving climate neutrality lies in their potential to reduce emissions in heavy industries that are considered hard to abate (i.e., where emission reduction is technologically very challenging) and their applicability to existing production facilities.

The primary carbon capture technologies are categorized based on the point at which the capture process occurs relative to combustion. These include pre-combustion capture, post-combustion capture, and oxy-fuel combustion capture [8]. Post-combustion capture is considered the most practical approach to reducing carbon dioxide emissions from the perspective of existing CO_2_-emitting installations. Pre-combustion and oxy-fuel combustion capture present challenges such as infrastructure requirements, severe corrosion, and high maintenance and servicing costs [9]. Post-combustion capture methods can be divided into adsorption, absorption, and membrane separation [9]. Currently, the most mature technology for capturing CO_2_ from industrial processes is absorption. In this method, CO_2_ molecules are dissolved in liquid solvents such as amines (monoethanolamine, diethanolamine, diethylenetriamine), hot potassium and potassium carbonates, chilled ammonia, and ionic liquids. Among these, amine-based systems are the most advanced and widely used [10]. However, according to [11], relying solely on a single technology based on chemical reactions for CO_2_ capture is impractical due to the enormous scale of existing emissions. Therefore, every developed and implemented technology contributing to CO_2_ emission reductions is essential. Currently, calcium looping is considered a critical technology for industrial decarbonization (though not fully commercialized), particularly in the cement industry [12]. This method is based on the reversible reaction of CaO with CO_2_ to form CaCO_3_, as illustrated in Equation (1) [13,14,15,16]:

CaO_(s)_ + CO_2(g)_ ⇌ CaCO_3(s)_ (1)

**Figure 1:**
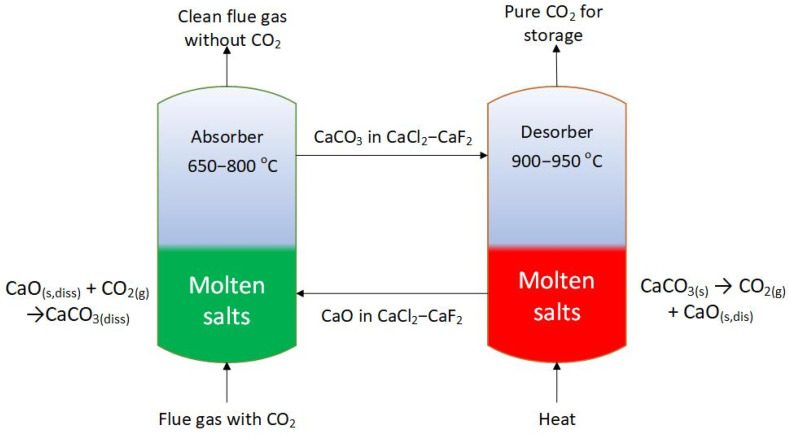
The concept of the carbon capture in molten salts process.

Thermodynamic data [17] for the above reaction indicate that the change in Gibbs free energy equals zero at 886 °C. At this temperature, the pressure of the CO_2_ reaches 1 atm. Since the reaction is reversible, its direction can be altered by raising or lowering the temperature, thus enabling the capture or release of carbon dioxide from the solid product. The captured carbon dioxide can then be transported to a storage site, used for Enhanced Oil Recovery, or converted into chemical feedstock. Carbon capture in molten salts (CCMS) is based on the same principles as CaL but with the active substances (CaO/CaCO_3_) dissolved or partly dissolved in molten salts. This approach allows for faster reaction kinetics and higher CO_2_ sorption capacity and helps avoid issues with solid attrition [18]. Moreover, CO_2_ absorption reactions are exothermic, which suggests that the capture process could be thermally self-sufficient. There is also the potential for heat recovery during the cooling of molten salts from the desorption process, which are then directed back to absorption. Energy consumption in the process is estimated at 8.8 GJ/tCO_2_ [19]. Importantly, the CCMS technology can be applied to existing CO_2_-emitting installations without interfering with their structure. The concept of the CCMS process is shown in Figure 1.

The process was experimentally investigated in a single-chamber reactor, involving the passage of an N_2_−CO_2_ (15 vol. % CO_2_) gas mixture through molten salts based on chlorides and fluorides [18,20,21]. Molten salts with a eutectic composition of CaF_2_−CaCl_2_ (13.8 wt. % CaF_2_ in CaCl_2_) demonstrated the most promising absorption and desorption properties [18]. This composition exhibited a higher carrying capacity value (0.599 g of CO_2_/g of CaO at 695 ÷ 705 °C) and complete decarbonation at 927 ÷ 947 °C [18].

In subsequent studies [22,23,24,25] related to the CCMS technology, the following were carried out:

- Evaluation of the impact of other compounds present in the gases on the capture process (SO_2_, N_2_, H_2_O);
- Optimization of the process to achieve maximum efficiency, absorption, etc.;
- Determination of the viscosity of molten salts and its potential impact on the process.

The effect of SO_2_ on the CCMS process was investigated in [22]. It was found that similar to the reactions of SO_2_ with CaO and CaCO_3_ in the solid state, sulfation reactions in molten salts are favored over carbonization reactions. Tests in a single-chamber reactor showed that almost all SO_2_ is absorbed by molten salts. The authors concluded that CCMS technology could be used to capture both CO_2_ and SO_2_ from flue gas, potentially avoiding the need for pre-desulfurization. However, it should be noted that the efficiency of the carbonization process might be slightly reduced. The regeneration of SO_2_-saturated salts should also be considered.

Olsen et al. [23] studied the effect of H_2_O on CaCl_2_-CaF_2_-CaO molten salts in terms of hydrolysis. The results indicated that hydrolysis occurs at elevated temperatures. Both salt components, CaF_2_ and CaCl_2_, form HF and HCl, respectively. However, the presence of CaO significantly reduces the hydrolysis process. It was found that hydrolysis is not a problem when significant amounts of CaO are present (10 wt. % or more) and at temperatures below 850 °C.

The study [24] identified the optimum temperature for the absorption process (approximately 700 °C) and the temperature at which CO_2_ release from the carbonate is fastest and complete, occurring at ~930 °C. A sustained CO_2_ capture capacity was observed after the initial 4 ÷ 6 carbonation/decarbonation cycles in all multiple tests. After 10 carbonation/decarbonation cycles, the CaO-CaF_2_-CaCl_2_ systems attained carrying capacity values of 0.667 g of CO_2_/g of CaO, respectively. A high absorption efficiency (>90%) was also observed in the first 2 h of the process for a CaO content of 15 wt. % of CaO. In the case of desorption, carbonate decomposition occurred with an efficiency of 100%. This shows that the dissolution/dispersion of CaO in molten CaF_2_−CaCl_2_ is a promising pathway for improving the reactivity and stability of CaO in long-term cyclic CO_2_ capture operation [24].

For the viscosity of the discussed molten salts, it was stated in [25] that up to 30 wt. % CaO in the eutectic CaCl_2_-CaF_2_ mixture within the temperature range of 700–950 °C, the viscosity should not pose a problem regarding the transport of molten salts.

The latest research was conducted at AGH University of Krakow (Poland) as part of a project in partnership with the Norwegian University of Life Sciences (Norway). This project focused on designing, constructing, and testing a reactor prototype operating in CCMS technology. The main design assumptions for the prototype were based on a structure consisting of two reaction chambers and an additional (auxiliary) tank, with the transport of molten salts forced by gas pressure in the respective tank (pumping). The design solution was patented [26] and described in studies [27,28]. All tanks, barbotage lances, connecting pipes, and valves were made from Nickel Alloy 200. A single reactor chamber was subjected to extended testing to verify the durability of the structural materials used and the feasibility of continuous operation. The purpose of this work was to evaluate the wear of the basic structural components, i.e., the barbotage lance and the reaction tank—components most exposed to prolonged exposure to molten salts CaCl_2_-CaF_2_-CaO-CaCO_3_, temperatures of 700 and 940 °C, and an N_2_-CO_2_ gas mixture.

## 2. Materials and Methods

### 2.1. Prototype Design

**Figure 2:**
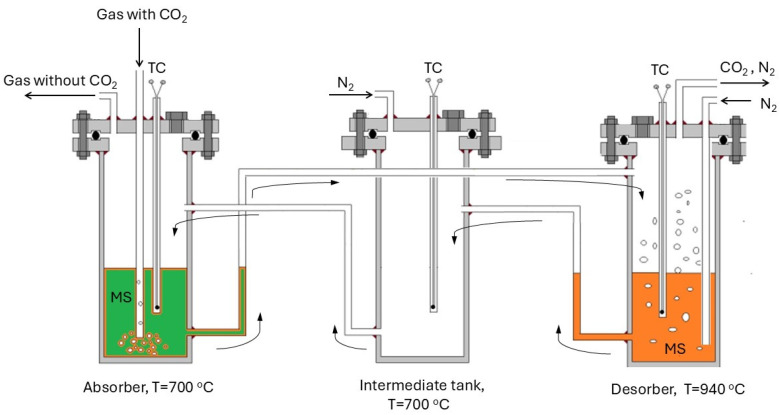
Diagram of the CCMS process using two chambers and an intermediate tank: TC—thermocouple, MS—molten salts CaCl_2_-CaF_2_-CaO-CaCO_3_.

Figure 2 schematically presents the CCMS process utilizing two reaction tanks (absorber and desorber) and an intermediate tank. The diagram also highlights the connection system between the tanks and the flow direction of the molten salts.

**Figure 3:**
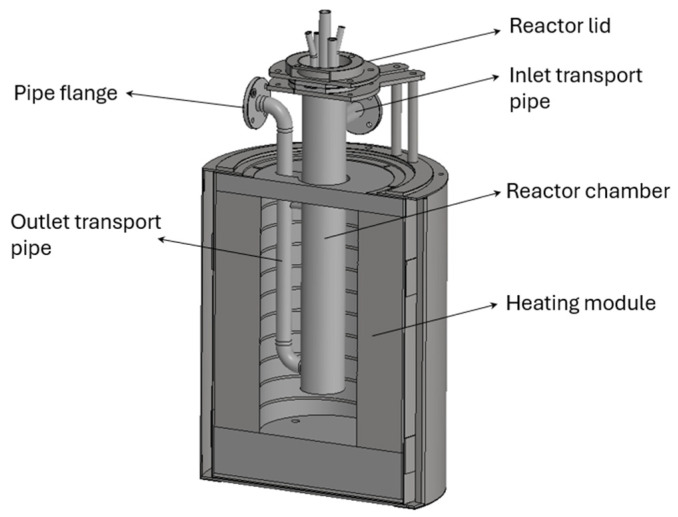
The 3D appearance of a reactor chamber with inlet and outlet transport pipes for molten salts placed within a heating module.

Figure 3 shows the basic structural elements of a single unit, identical for the absorber, desorber, and intermediate tank. The reactor chambers, along with the transport pipes (horizontal inlet and vertical outlet), were placed within heating modules and closed with tight lids. Gases were introduced and extracted through openings located in the lids. One opening was also left for inserting a thermocouple.

**Figure 4:**
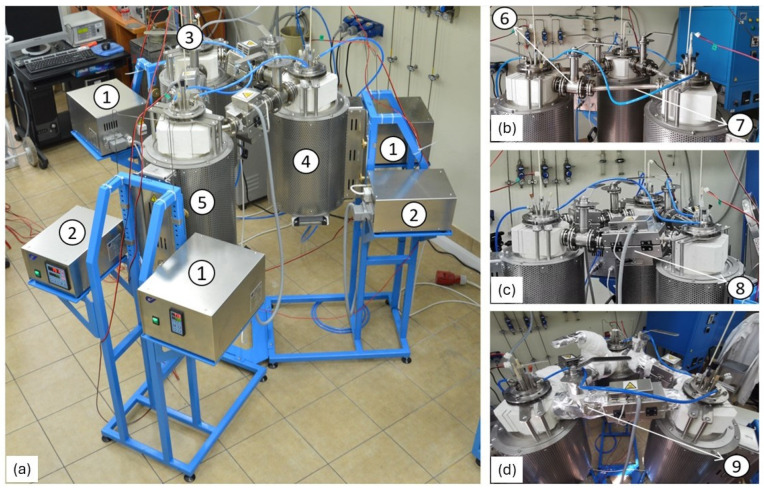
View of the prototype reactor for carbon capture at the AGH laboratory, (**a**) overall view: 1, 2—power supplies; 3—absorber; 4—desorber; 5—intermediate tank, (**b**) view of the system without additional heating modules: 6—high temperature valve; 7—transporting pipe, (**c**) view of the system with additional heating modules: 8—heating module; (**d**) view of the system with valves insulation: 9—ceramic wool insulation.

The chambers were connected to each other by horizontal transport pipes, as shown in Figure 4b.

On each transport line between the tanks, there was a high-temperature, gas-tight valve (Figure 4b). This valve allowed the transport line to be shut off, forcing the flow of molten salts to the next tank. To maintain appropriate temperature conditions during salt transport, additional heat sources in the form of removable electric furnaces were installed on the horizontal pipes (Figure 4c). The high-temperature valves were insulated with ceramic mats (Figure 4d). Each reactor chamber heating module, as well as the furnaces installed on the horizontal transport lines, had their own power supply sections (Figure 4a). The construction principle of the prototype is described in detail in studies [27,28].

### 2.2. Prototype Operation Methodology

The prototype has been designed to operate cyclically [26], with the absorber and desorber working in parallel, aiming for maximum utilization of the active sorbent and complete regeneration of the salts. The operational cycle of the prototype will begin by filling the absorption tank and the intermediate tank with salts of the same composition, containing an eutectic mixture of CaCl_2_-CaF_2_ with a 15 wt. % of CaO. After melting the salts, the absorption chamber will initiate the process of capturing carbon dioxide from an N_2_-CO_2_ mixture, injected into the salts using a lance. This process will continue until the full utilization of CaO (when the CO_2_ concentration in the gases leaving the reactor reaches the level concentration as in the mixture injected into the salts). At this point, the molten salts will be transported to the desorber and preheated to 940 °C. The transport will be carried out by pumping the salts using an inert gas (N_2_) injected into the reactor chamber.

After transferring the CO_2_-saturated salts to the desorber, the calcination process of calcium carbonate and the release of CO_2_ will begin. Simultaneously, the unsaturated salt from the intermediate tank will be transported to the absorber, where the CO_2_ capture process from the N_2_-CO_2_ stream will restart. In this way, the processes of CO_2_ capture and release will proceed simultaneously in both tanks (the absorber and the desorber). Upon completion of these processes, the transport of salts will take place first from the desorber to the intermediate tank and then from the absorber to the desorber, initiating the next operational cycle. The reactor chambers will operate under atmospheric pressure during the absorption and desorption processes. Pressure will increase only during the transport of salts between the chambers. The pumpability tests of the salt showed that a pressure of approximately 0.4 atm will be required.

### 2.3. Testing Conditions for a Single Reactor Chamber

**Figure 5:**
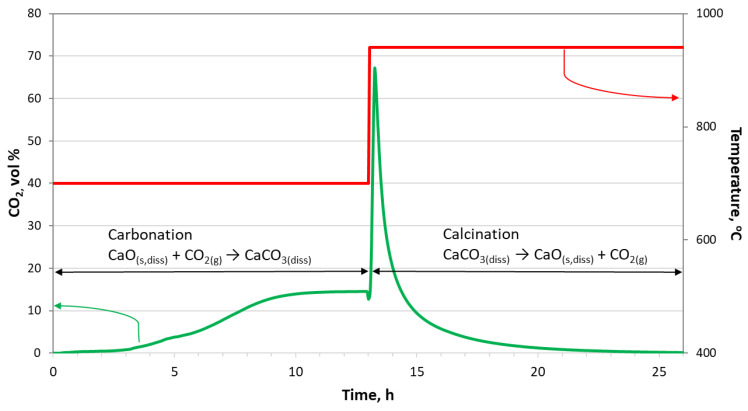
Changes in carbon dioxide concentration during a single, complete operating cycle of the CO_2_ capture reactor chamber (green line) and process temperature (red line).

One of the reaction chambers was subjected to high-temperature tests, operating in a cyclic manner with periodic temperature changes. The tests on a single chamber aimed to determine the impact of aggressive operating conditions (rapid temperature changes, the presence of chloride–fluoride salts, continuous gas flow, changes in the chemical composition of salts during the process) on the wear of its key working components—the reaction chamber and the lance. At a temperature of 700 °C, CO_2_ capture from the CO_2_/N_2_ gas mixture (15 vol. % CO_2_) occurred for approximately 13 h. Then, the temperature was raised to 940 °C, and with a pure nitrogen flow, the previously absorbed CO_2_ in the salts was released. The desorption time was also 13 h. The salts (total mass 800 g) contained a eutectic mixture of CaCl_2_-CaF_2_ (13.8 wt. % CaF_2_) and 15 wt. % CaO. During absorption, the N_2_-CO_2_ gas mixture flowed through the salts at a rate of 24 dm³/h. Pure nitrogen flowed at the same rate during desorption. The salt level in the reactor was approximately 13 cm. During the capture cycle (700 °C), the CaO concentration decreased as calcium carbonate (CaCO_3_) formed according to the carbonation reaction (1). During desorption (940 °C), reaction (1) proceeded in the opposite direction, resulting in a decrease in CaCO_3_ content and an increase in CaO concentration. An example graph of changes in carbon dioxide concentration during a single, complete sorption (carbonation)—desorption (calcination) cycle is shown in Figure 5.

Similar changes in temperature and CO_2_ concentration occurred in each cycle during the 800 h of reactor operation. For the first 3–3.5 h, the process operated with very high efficiency, above 96%. This meant that at least 96% of the CO_2_ introduced into the molten salts was absorbed as CaCO_3_. After this time, due to the limited availability of CaO in the salts, the absorption efficiency decreased. The complete depletion of CaO was indicated by the plateau on the curves. On the other hand, the calcination process (release of CO_2_) operated at a minimum of 97% efficiency. The CO_2_ capture capacity of the prototype was 0.0031 mol/min. The reactor chamber was monitored 24 h a day, particularly the temperature, composition, and flow rate of the N_2_-CO_2_ gas mixture introduced into the reactor, as well as the composition of the gases leaving the reactor. During the experiments, the reactor chamber operated without issues requiring staff intervention, such as interruption of the experiment, shutdown of the equipment, cooling of the reactor chamber, replacement of salts, etc.

### 2.4. Materials Subjected to Testing

#### 2.4.1. Characteristics of Ni 200 Alloy

The reactor chambers and the lances introducing the N_2_-CO_2_ gas mixture, as noted in Section 1, are made of technical nickel (Ni 200 Alloy), whose composition is presented in Table 1.

It is highly resistant to corrosive environments, especially caustic alkalis, acids, and salt solutions. It also exhibits high thermal and electrical conductivity. The use of technical nickel as the construction material for the main components of the CCMS reactor prototype, whose elements were tested in this study, was mainly motivated by three reasons:

- Nickel 200 alloy has been used as a material for crucibles and lances in all previous experiments at NMBU [19,20,21,22,23,24,25], where the CCMS process was tested under various temperature conditions (650–950 °C) and in the presence of molten salts with different compositions (chlorides, fluorides, oxides, carbonates);
- The complexity of the prototype constructed under laboratory conditions required materials suitable for forming individual parts through plastic deformation processes and allowing easy welding to maximize protection during continuous, 24 h high-temperature tests;
- The use of other nickel alloys would have been risky, as alloying elements (mainly Cr) present in nickel/chromium alloys, Inconel, and Hastelloy [29,30,31,32,33,34] significantly accelerate the rate of corrosion, and grain boundaries are preferred sites for corrosion attack [35,36].

#### 2.4.2. Samples for Testing

**Figure 6:**
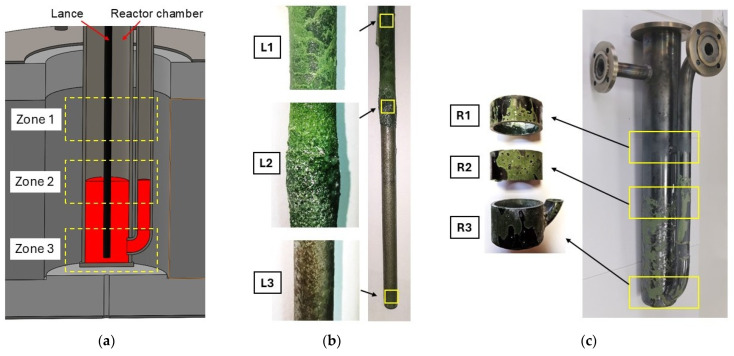
View: (**a**) cross-sectional view of the reactor chamber with marked zones with different operating conditions, (**b**) lance after 800 h tests with marked zones subjected to analysis, (**c**) reactor chamber after 800 h tests with marked areas and cut-out sections subjected to analysis.

After the long-term tests, the molten salts were poured into a separate container, and both the lance and the reactor chamber were cooled. Then, to remove residual crystallized salts, the metal components were submerged in a container filled with distilled water for 24 h, with the water changed three times. After drying, the reactor was subjected to an X-ray examination. In the next stage, elements of appropriate size were cut from three zones of both the reactor and the lance using a bandsaw and were examined using computed tomography (CT), 3D scanning (reactor), and microscopic analysis (lance). Figure 6 shows the cross-sectional view of the reactor chamber with marked zones with different operating conditions, and samples of lance and chamber reactor subjected to analysis.

The elements marked in Figure 6b,c came from three zones:

- Zone 1: Gas and temperature exposure zone (elements R1, L1). For the reactor tube, this involved continuous exposure to the oxidizing atmosphere on the outer side and the N_2_-CO_2_ gas mixture on the inner side. During a complete sorption–desorption cycle, the composition of the gas mixture varied according to the curve shown in Figure 5. The outer part of the lance was subject to similar conditions, while the inner side was exposed to the N_2_-CO_2_ mixture during the low-temperature absorption cycle and pure N_2_ during the high-temperature desorption cycle. Contact with splashing molten salts due to sparging could not be ruled out. Due to the temperature gradient at the height of the reactor chamber above the molten salts level, the temperature was slightly lower than in the lower zones of the tank, reaching about 650 °C during sorption and 900 °C during desorption.
- Zone 2: Phase boundary zone between molten salts and N_2_-CO_2_ gases (elements R2, L2). The contact of both the lance and the salts was similar to that described above; however, it is difficult to precisely determine the exposure time of the elements to salts and gases due to the high intensity of sparging, which caused the molten salt surface to be in constant motion (rising, falling, and turbulent). The temperature in this zone was 700 °C/940 °C.
- Zone 3: Filled with molten salts (elements R3, L3). In the case of the reactor tube, the inner part and the bottom were exposed continuously to the molten salts, while the outer part was exposed to the oxidizing atmosphere (air) in the space between the heating module and the reactor tube. The lance, on the other hand, was exposed to molten salts on its outer side to the N_2_-CO_2_ gas mixture on the inner side during the low-temperature absorption cycle and to pure N_2_ during the high-temperature desorption cycle. The temperature in this zone was 700 °C/940 °C.

**Figure 7:**
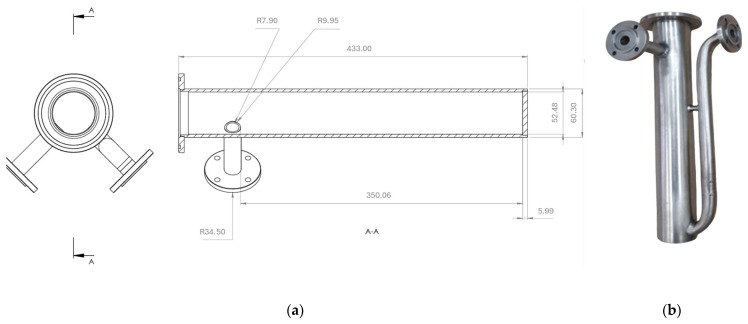
Reaction chamber: (**a**) main initial dimensions (before experiments), in mm, (**b**) appearance before testing.

The appearance of the reactor chamber before testing and its main initial dimensions are shown in Figure 7.

In the case of the lance, its internal diameter was 5.6 mm, the outer diameter was 9.8 mm, and the length was 1000 mm.

### 2.5. Methods of Analysis

In assessing the wear of the reactor chamber, several testing methods were used (X-ray, CT, 3D SCAN) based on several key criteria:

- Direct evaluation of changes in geometric dimensions after testing;
- Identification of internal discontinuities in structural components (defects, voids, pores), quality of welds;
- Detection of wear inhomogeneity, localized pits, and cracks;
- Evaluation of the suitability of individual methods for this type of analysis.

Moreover, the use of X-rays allowed a complete assessment of the reactor without the need to cut it into separate components. X-ray computed tomography, in addition to multi-plane analysis, allowed for the evaluation of wear inhomogeneity across different zones. The 3D scanning was employed as a supplementary technique and made it possible to locate deviations (both positive and negative) from the nominal values of the reactor chamber.

#### 2.5.1. X-Ray

The research utilized the Zeiss BOSELLO 2D X-ray Solutions system (Carl Zeiss IQS Deutschland GmbH, Carl-Zeiss-Straße 22, 73447 Oberkochen, Germany). ZEISS BOSELLO systems are 2D X-ray cabinets equipped with software for Automatic Defects Recognition (ADR) (Software BHT IP Plus). The dedicated software enabled fast image acquisition and fully automated 2D X-ray inspection in accordance with ASTM standards [37].

During the tests, a maximum source voltage of 225 kV and a maximum power of 1800 W was applied, with a detector resolution of 1024 × 1024 and a pixel size of 200 µm. The inspection time was 1 min, and the time for a single image capture was 200 ms.

The entire reactor chamber was subjected to X-ray examination after the tests.

#### 2.5.2. Computed Tomography (CT)

The elements of the reaction chamber were scanned using the GE Phoenix v|tomex|m X-ray tomography system (Waygate Technologies USA, LP 721 Visions Dr, Skaneateles, NY 13152, USA). The object was inspected in a specialized chamber of the device. The element was positioned between the radiation emitter, known as the lamp, and the detector. The device was controlled by setting the position of the object in the space between the lamp and the detector, entering the voltage and current values generating the radiation (power), and specifying the detector’s operating characteristics. Proper selection of these parameters allowed for results that enabled further 3D reconstruction of the object.

The next step was the reconstruction of the 3D volume. During a full rotation, the device generated thousands of high grayscale images (e.g., the GE Phoenix v|tomex|m uses 14-bit). It is standard to use complex algorithms in the reconstruction stage that perform image corrections, such as beam hardening correction, automatic geometry calibration, and geometry optimization [38,39]. The reconstructed object can be visually inspected through the analysis of 2D and 3D cross-sectional images and further volumetric analysis.

It should be noted that all measurements were conducted with identical detector settings, voltage, and consistent positioning of the maneuvering table. The results were obtained based on reconstruction with the same algorithm: using the bhc+ filter (coefficient 7.1), auto AGC, and reconstruction optimization. Determining porosity (voids, empty spaces) is widely used when analyzing heterogeneous materials [40,41] or changes in materials due to external factors [42]. The examination of a single reactor tube element consisted of 3000 X-ray projections. The size of a single voxel did not exceed 60 µm. Radiation was generated by a microfocus lamp with a 260 kV and 190 µA characteristic. During reconstruction, a beam hardening correction+ filter with a power of 7.1 and automatic geometry correction were applied.

The reactor components R1, R2, and R3 were subjected to computed tomography.

#### 2.5.3. Scan 3D

A setup consisting of a GOM 3D scanner measuring head (GOM, a Zeiss company, Oberkochen, Germany), a tripod, a movable table, a calibration plate, and a portable Dell computing unit was used for optical 3D scanning.

The ATOS Core 200 scanner head ( GOM a Zeiss company, Oberkochen, Germany) used for measurements was equipped with two 5 MP cameras, blue LED lighting, and light with a wavelength range from 450 to 500 nm. The measurement accuracy, verified according to standard [43] and confirmed by an accredited, independent metrology laboratory, is 0.0185 mm. The time required to complete a single scan was less than 2 s. The research was conducted using Atos Scan 2017 software, which enabled the scanning process and the creation of a 3D model, as well as previously described GOM Inspect software for 3D measurement data analysis (2019 Hotfix 6 Rev. 125216). The guidelines for assessing the accuracy and acceptance of optical 3D scanning systems are contained in the standard [44] and the recommendations of the German PTB in accordance with the standard [43].

The reactor component R3 was subjected to 3D scanning.

#### 2.5.4. Microstructural Studies

For microstructure analysis, an Olympus GX51 light microscope (OLYMPUS GX51 microscope, Tokyo, Japan) and a Hitachi SU 70 scanning electron microscope (Hitachi Ltd., Tokyo, Japan) equipped with energy-dispersive X-ray spectroscopy (EDS) detector for chemical composition analysis in micro-areas were used (Thermo Fisher Scientific, Waltham, MA, USA). Images were captured using the secondary electron (SE) and backscattered electron (BSE) detectors. Samples for microscopic examination were embedded in PolyFast conductive resin (Struers, Copenhagen, Denmark), then ground using abrasive papers with the following grit sizes: 220, 500, 800, 1200, 2400, and 4000, and polished in two stages according to the Struers procedure using Dia Duo diamond paste suspension (Struers, Copenhagen, Denmark) with a 3 µm grain size and OPS colloidal silica suspension with a 0.25 µm grain size for final polishing. The samples were not etched.

The lance components L1, L2, and L3 were subjected to microstructural analysis.

## 3. Results

### 3.1. Barbotage Lance Tests

**Figure 8:**
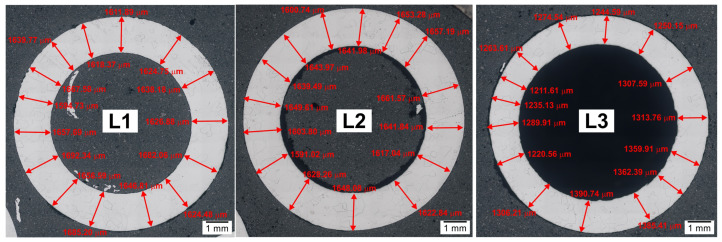
Appearance of samples L1, L2, and L3 and wall thickness measurement points (light microscopy—macro image).

The results of the lance wall thickness measurements using light microscopy at various points across the cross-section of the examined samples are presented in Figure 8. Table 2 summarizes the minimum, maximum, and average values, as well as the calculated wall thickness reductions (thinning).

The obtained wall thickness results on the cross-section of the examined lance demonstrated thinning relative to the original dimensions. The degree of thinning depends on the lance’s operating zone. The average wall thickness on the lance cross-section before experiments was 1647 µm, with wall thickness variations across the cross-section reaching up to 62 µm. The greatest wall thinning after high-temperature prototype tests was observed for the element labeled “L3” (continuous external contact with molten salts CaCl_2_-CaF_2_-CaCO_3_-CaO, internal contact with N_2_-CO_2_ gases). In this case, the wall thinned by an average of 353 µm, or 21.43%. Significant variations in wall thickness across the cross-section were also found, reaching up to 180 µm locally.

For samples labeled “L2” and “L1”, slight wall thinning of the lance was observed, averaging 10 µm (0.61%) for element L2 and 2 µm (0.12%) for element L1. Wall thickness differences across the cross-section reached up to 70 µm for element L2 and 100 µm for element L1.

**Figure 9:**
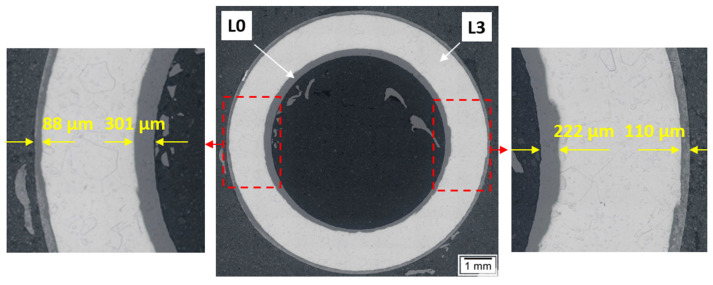
Images of the L3 lance section after testing and before testing (L0). A 50% image transparency was applied for L0.

When analyzing the L3 section of the lance, it was compared to the material before testing. Images from the light microscope were superimposed, with 50% transparency applied to the sample before testing—L0 (Figure 9).

It was found that the lance experienced significantly greater wear on the inner side. The wear is uneven around the circumference on both the outer and inner sides of the lance.

**Figure 10:**
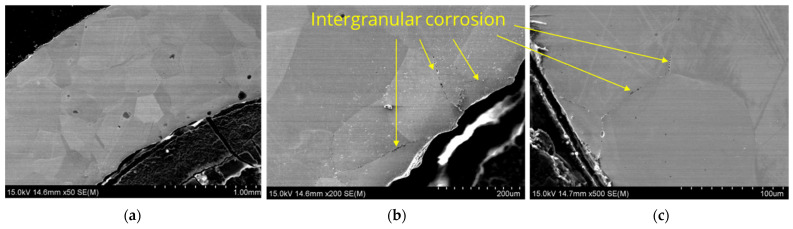
Microstructure images of sample L3, SEM: (**a**) magnification 50×, (**b**) inner section, magnification 200×, (**c**) outer section, magnification 500×.

**Figure 11:**
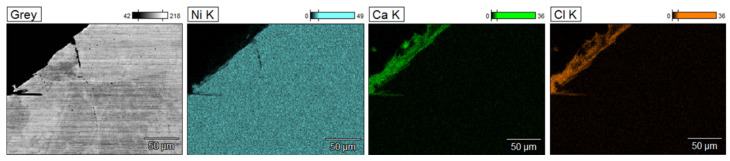
Ni, Ca, Cl distribution maps of sample L3; SEM/EDS.

Figure 10 shows example results of the microstructure analysis of sample L3 performed using a scanning electron microscope, while Figure 11 presents the results of chemical composition analysis in micro-areas using an EDS detector.

The microstructure observations on the cross-section of the tube labeled L3 demonstrated the presence of intergranular corrosion on both the inner and outer sides of the tube. The corrosion spreads from the outer and inner surfaces of the lance inward, locally reaching a depth of several hundred micrometers. Corrosion appears as a network of voids spreading along grain boundaries. The SEM/EDS chemical composition analysis results indicate that these areas are nickel-depleted (Figure 11), and some spaces are infiltrated by salt components (Ca, Cl). Salt components were also detected on the analyzed outer surface, which are residues of crystallized salts that did not dissolve in water during lance rinsing.

**Figure 12:**
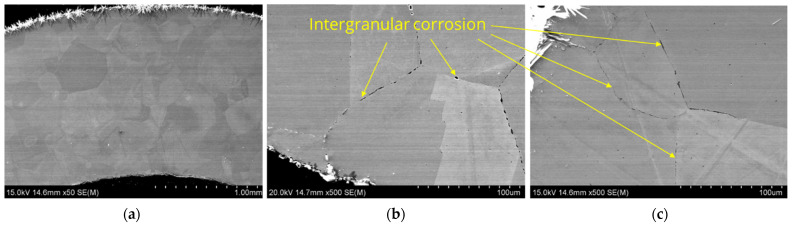
Microstructure images of sample L2, SEM: (**a**) magnification 50×, (**b**) inner section, magnification 500×, (**c**) outer section, magnification 500×.

**Figure 13:**
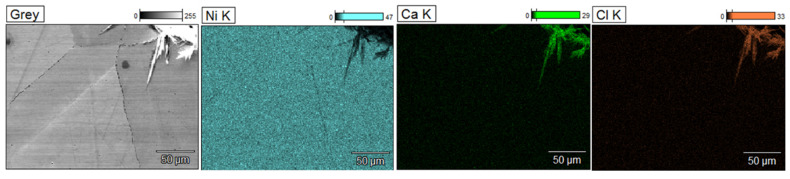
Ni, Ca, Cl distribution maps of sample L2; SEM/EDS.

**Figure 14:**
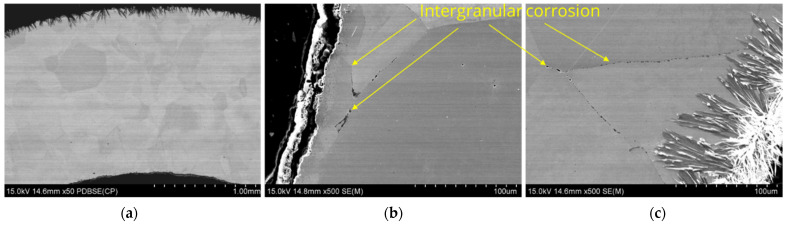
Microstructure images of sample L1, SEM: (**a**) magnification 50×, (**b**) inner section, magnification 500×, (**c**) outer section, magnification 500×.

**Figure 15:**
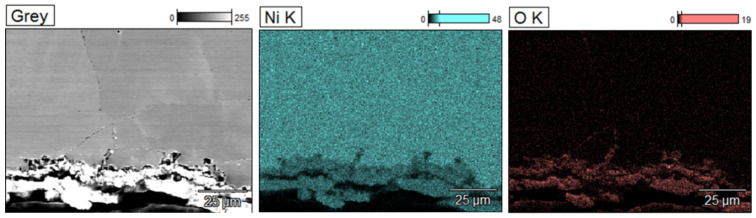
Ni, O distribution maps of sample L1; SEM/EDS.

Similar microscopic observations and analyses were conducted for samples taken from the other two zones, labeled L2 and L1. The results are presented in Figure 12, Figure 13, Figure 14 and Figure 15.

**Figure 16:**
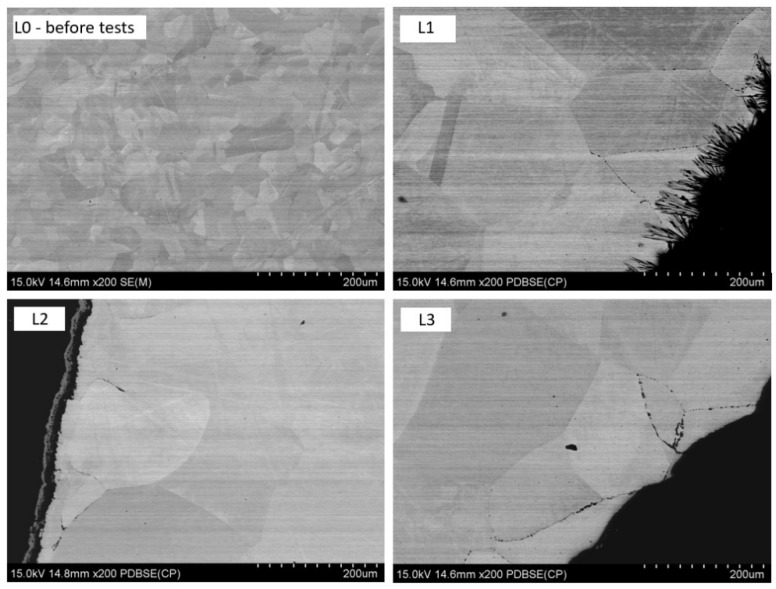
Comparison of grain size in Ni Alloy 200 before testing (L0) and after testing (L1, L2, L3).

**Figure 17:**
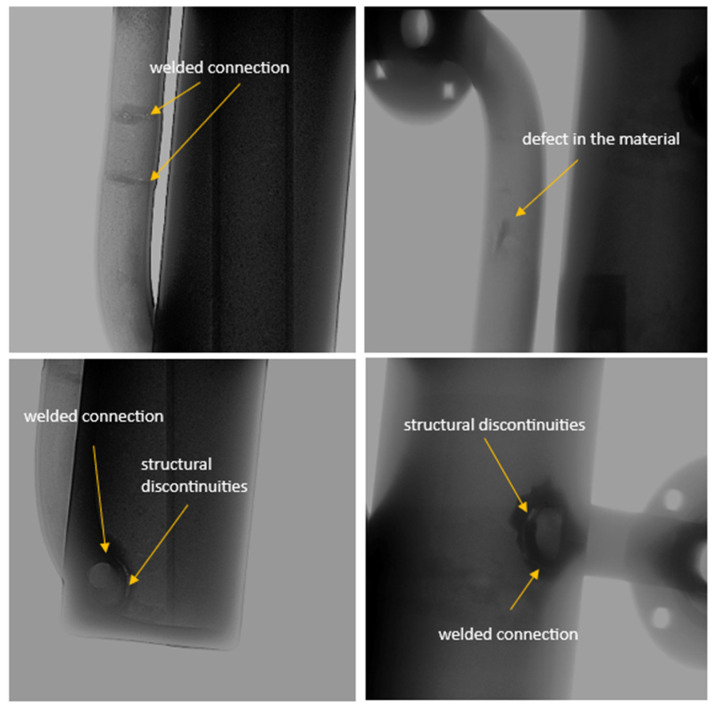
Selected inspection photos of the reactor chamber after testing.

Analyzing the results presented in Figure 14, Figure 15, Figure 16 and Figure 17, it was observed that corrosion appears both as a network of fine voids spreading along grain boundaries and as a continuous network at grain boundaries. The SEM/EDS chemical composition analysis results indicate that these areas are nickel-depleted. Additionally, the images show residues of crystallized salts that formed on the sample surfaces and were not dissolved in water after the experiments.

Figure 16 presents a comparison between the original Nickel 200 Alloy samples and the samples after testing.

**Figure 18:**
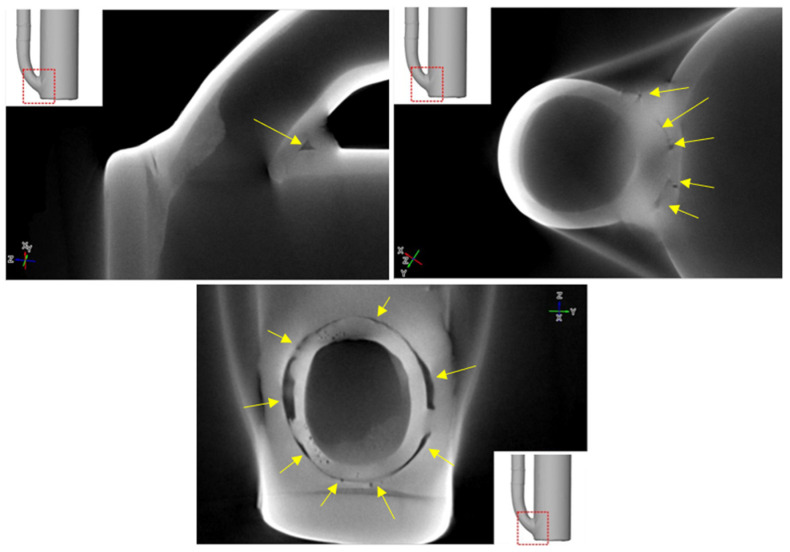
Analysis of the lower part of the reactor chamber after reconstruction to the 3D model. Yellow arrows indicate voids and discontinuities in the material.

Analyzing Figure 18, it can be observed that the microstructure of the initial tube cross-section, labeled L0, is uniform and consists of grains with regular shapes, averaging about 50–60 µm in size. No precipitates or discontinuities are visible within the examined element. In samples L1–L3, significant grain growth was observed in each tested sample after the tests, regardless of the presence or absence of molten salts or N_2_-CO_2_ gases. Some grains exceeded 200 µm in size. In each case, the material was exposed to high temperatures; due to the temperature gradient along the reactor, samples L3 and L2 were subjected to temperature fluctuations between 700 and 940 °C, while sample L1 experienced slightly lower temperatures (650/900 °C).

### 3.2. X-Ray Examination of the Reactor Chamber

The X-ray inspection allowed the detection of defects, damage, and discontinuities in the structural material without the need to cut the reactor into smaller components. Figure 17 shows selected inspection images of the reactor chamber after inspection.

After X-rays were taken, reconstruction to a 3D model was performed and the lower part of the reactor chamber was analyzed in detail (Figure 18).

Darker areas marked with yellow arrows in Figure 17 and Figure 18 are local density changes in the analyzed material. Figure 17 shows welded joints and a defect in the material, probably formed during the manufacture of the reactor tube. Most of the identified defects (voids, material discontinuities) are due to the inability to weld structural elements of the chamber from the inside of the reactor. They are not a result of reactor operation or prolonged exposure to the corrosive environment. However, the obtained information is important from the perspective of system sealing and properly securing the chamber against leakage of molten salts at a temperature of 700/940 °C into the heating module, which could lead to a failure of the entire device.

### 3.3. CT Scan Results

**Figure 19:**
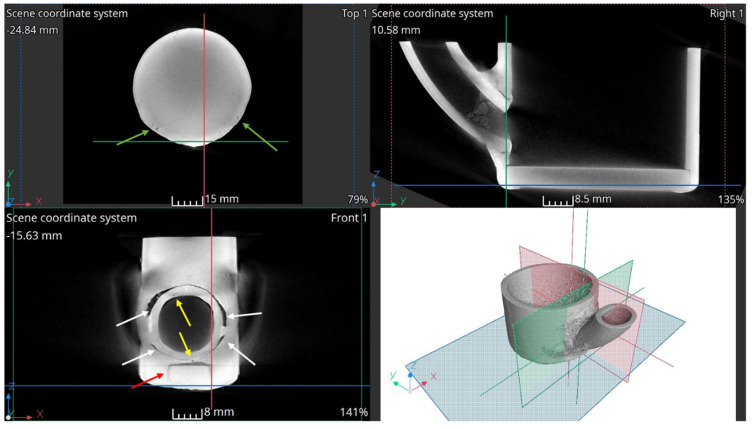
Cross-sectional images of the element R3.

X-ray computed tomography used for the analysis required cutting the reactor components into smaller sections. The study examined reactor fragments operating in three zones, as described in Section 2.1. Figure 19 shows selected cross-sectional images of the lower part of the reactor chamber (R3).

White arrows indicate defects (lack of full remelting) in the joint between two pipe elements. Lack of remelting occurs around the entire circumference at the junction of the smaller-diameter pipe with the reactor pipe (larger diameter). In addition to the lack of full penetration, voids in the weld and numerous pores, marked by yellow arrows, are also visible, some of which form larger clusters. The connection between the reactor pipe and the flat base is also visible. Here, too, there is incomplete remelting (red arrows) and clusters of pores, marked by green arrows. The tricolor lines on the cross-sectional images indicate the positions of the other cross-sections corresponding to the viewing planes shown in the 3D section.

**Figure 20:**
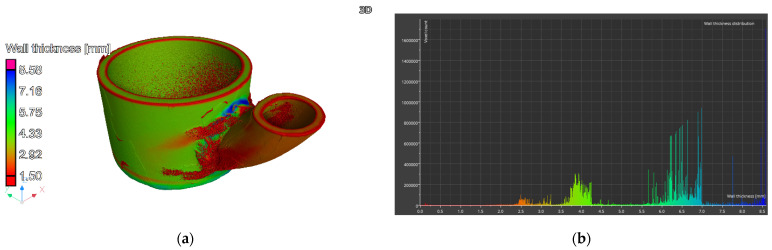
Element R3: (**a**) 3D view and (**b**) wall thickness distribution.

A wall thickness analysis of the R3 element was performed on the reconstructed data. The presented model, created using computed tomography, exhibits noise primarily resulting from the complexity of the shape and geometry of the analyzed component (Figure 20a).

Artifacts characteristic of this method, such as data-disrupting peaks, hinder the direct interpretation of the results. Nevertheless, the application of advanced filtering procedures has partially mitigated these disturbances, as confirmed by the provided graph. Thickness distribution analysis is further complicated by the presence of noise in the visual representation and the variable thickness of individual components, which becomes apparent when analyzing the data depicted in the graph (Figure 20b). A particular challenge is the attachment of components via welding, which is characterized by uneven thickness on the weld surface, rendering the analysis of wear in specific areas unreliable.

**Figure 21:**
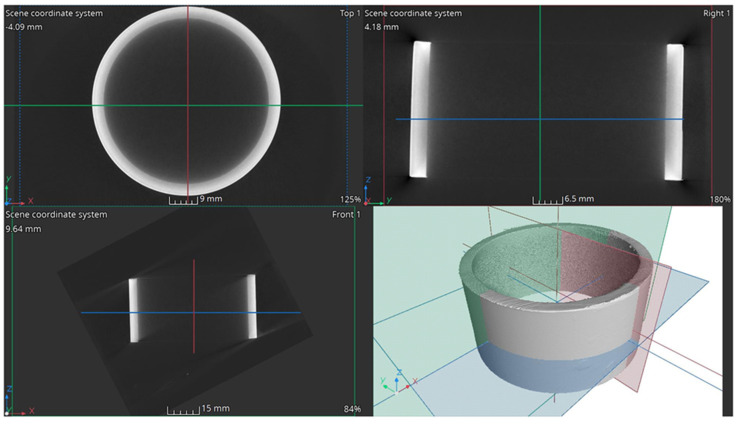
Cross-sectional images of the element R2.

In the case of the R2 element, which has partial contact with molten salts and a gaseous atmosphere, no irregularities, defects, or deep pitting were found in the cross-sectional images presented in Figure 21. The tricolor lines on the cross-sectional images indicate the positions of the other sections, corresponding to the viewing planes shown in the 3D section.

**Figure 22:**
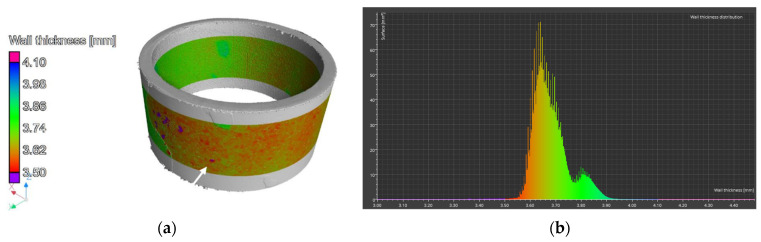
Element R2: (**a**) 3D view and (**b**) wall thickness distribution.

A wall thickness analysis of the R2 element was performed on the reconstructed data. The results of the analysis are shown in Figure 22.

The color scale indicates the wall thickness, which in this area is 3.75 mm. Areas of thickening, marked with a white arrow, are clearly visible in the figure, with a thickness of 3.82 mm. The section of the pipe shown in Figure 22a is noticeably thinner. Around the circumference of the element, there are spots colored in shades of red, indicating localized thickness reduction. The thickness change is approximately 0.2 mm. Figure 22b presents a thickness distribution graph of the element. Two peaks are visible—one at a thickness of 3.75 mm and the other at 3.82 mm. These values correspond to the visually identified thicknesses. The wall thickness in 99% of the entire analyzed region falls within the range of 3.51 mm to 3.945 mm.

**Figure 23:**
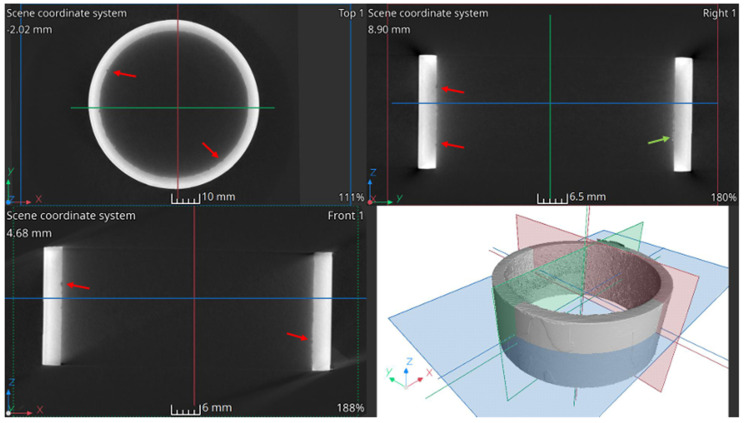
Cross-sectional images of the element R1.

Cross-sectional images for element R1 are shown in Figure 23. Their analysis indicates the presence of pitting on the inner side of the element, marked by red arrows. Additionally, a deposit is visible on the inner side (green arrows), which may be the result of crystallized and insufficiently flushed salts.

**Figure 24:**
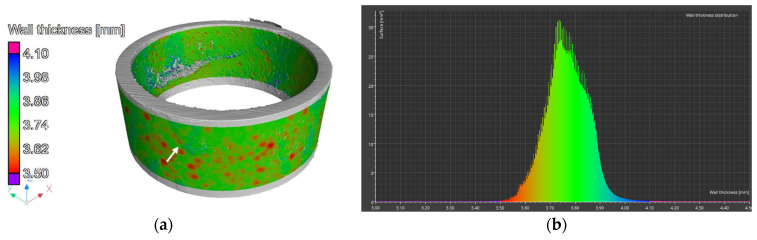
Element R1: (**a**) 3D view and (**b**) wall thickness distribution.

A wall thickness analysis of the pipe element was performed on the reconstructed data. The analysis results are shown in Figure 24. The thickness of element L1 is approximately 3.73 mm. In the 3D images, areas of thickening are clearly visible, marked with a white arrow. Around the entire circumference of the element, there are red-colored spots, indicating localized thickness reductions. The thickness variation is approximately 0.25 mm.

### 3.4. 3D Scanning Method

**Figure 25:**
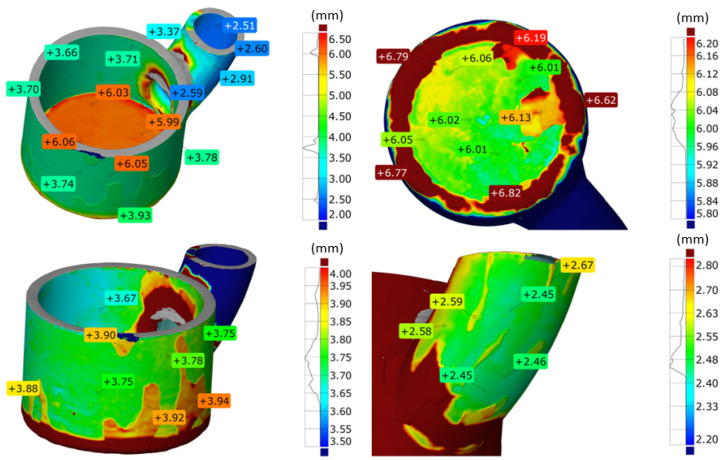
Thickness measurement of element R3 using the 3D scanning method.

Due to difficulties in assessing the thickness of element R3 using computed tomography, a thickness analysis was conducted based on 3D scans. The results of this analysis are presented in Figure 25.

For the analysis, it was necessary to adjust the dimension scale to the analyzed fragments. When presenting the results for the entire element, a significant number of thickness values were observed within the ranges of 2.2–2.8 mm, 3.5–4 mm, and 5.8–6.2 mm. These values, visible on the graph presented alongside the measurement scale, correspond to the nominal thicknesses of the bottom, the lateral part of the element, and the welded component of the device. The bottom of the element exhibits a thickness of approximately 6 mm, with significant irregularities resulting from the joining method (welding). The majority of results fall within the range of 5.95 mm to 6.12 mm. Defects on the external surface in the form of uneven corrosion are visible. Additionally, the inner surface shows isolated thickened areas caused by salt residues present in the device during operation. The cylindrical section also exhibits wear due to corrosion, visible on the external surface as large areas with varying thicknesses. The thickness difference averages 0.15 mm. The majority of results fall within the range of 3.65 mm to 3.8 mm. The welded component is characterized by significantly lower thicknesses, ranging from 2.4 mm to 2.6 mm. It contains areas with variable thickness and an elongated shape, which are also the result of corrosion-induced wear. The thickness difference averages 0.14 mm. The weld area was not analyzed due to significant thickness variations resulting from the welding technology itself.

**Figure 26:**
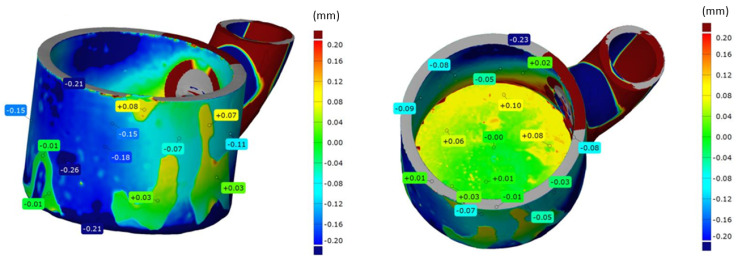
Lower part of reactor chamber (R3) shown from two views—dimensional comparison with CAD model.

The dimensional comparison of the R3 element after the tests with the CAD model is presented in Figure 26.

The deviations obtained in the working part of the component range from +0.08 mm to −0.26 mm. No significant deformation was observed in the cylindrical area of element R3. A comparison of the scanned element with the CAD model showed a significant shift of the welded element, but this did not affect the device’s performance. This shift likely did not result from the prolonged operation of the reactor chamber but rather occurred during the joining (welding) of the elements. The inner surface of the cylindrical area shows minimal wear, with mostly positive values at measurement points, suggesting slight wear and the deposition of molten salts, especially in the lower areas of the reactor (at the bottom). Wear primarily occurred on the outer part of the element, where values reach −0.26 mm. Wear is not uniform, with large areas showing no wear and small localized areas with increased wear, which may suggest a significant influence of corrosion due to operating conditions (mainly high temperature and an oxidizing atmosphere).

## 4. Discussion

The use of molten salts in the carbon dioxide capture process is a promising and highly efficient technique for CO_2_ reduction from diluted industrial gases. One of the greatest challenges of this new technology is finding construction materials with adequate corrosion resistance to chloride–fluoride molten salts and high temperatures (up to 940 °C). The corrosion of materials is caused by the high activity of ions in molten salts, especially at elevated temperatures [45]. This phenomenon is only partially understood, and the mechanisms remain unclear [46,47,48,49,50]. Nickel and its alloys are among the best materials for structural applications in molten salts, as they exhibit very good corrosion resistance and favorable mechanical properties [51,52,53].

From the perspective of the reactor’s long-term operation (with three chambers), the evaluation of the main structural components was the most crucial. In the case of the prototype, these components were the lance and the reactor tank. These elements will be subjected to the highest levels of corrosive environmental stress (molten salts and temperature) during continuous operation. Analysis of the microscopic observations of the lance revealed that wear varied depending on the zone in which the lance material was exposed to specific conditions. Measurements of the lance wall thickness before and after testing allowed for a direct assessment of its wear. The change in wall thickness in the lowest zone (continuous contact with molten salts) was approximately 21.4%. The lance in the intermediate zone (liquid/gas phase boundary) showed a slight thinning, as did the section operating above the molten salts, with thinning in these zones measuring 0.6% and 0.1%, respectively. Based on the results relative to the material’s exposure time in molten salts, it can be concluded that in the area most exposed to corrosion, the wear rate of Ni Alloy 200 was 353 µm/800 h, which translates to 3.9 mm/year. For the middle section of the tube, not directly exposed to molten salts, the wear rate was 109 µm/year, while the upper part showed a wear rate of approximately 23 µm/year.

**Figure 27:**
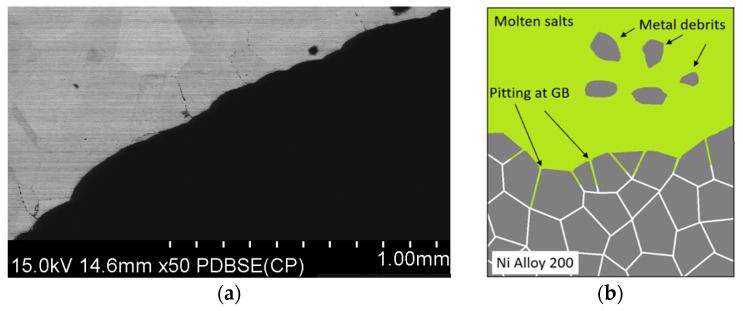
SEM image of the L3 surface (**a**) and schematic illustrating the formation of a pore-salt network for Ni Alloy 200 and molten salts (**b**).

Analysis of the lance thickness changes for section L3 showed uneven wear around the lance circumference and significantly greater wear on the inner side compared to the outer side. It was expected that higher wear would occur on the surface exposed to continuous contact with molten salts rather than in the gaseous atmosphere (N_2_-CO_2_). It is possible that molten salts were drawn into the end part of the lance, which was analyzed. In the analyzed cross-section of sample L3, characteristic pits were also observed, whose shape suggests the detachment of entire grains due to the potential penetration of molten salts through grain boundaries, as illustrated in the diagram in Figure 27.

The reaction of Ni with gases that may form in the salt environment, such as HCl or HF produced in reactions (2) and (3), also cannot be ruled out.

CaCl_2(l)_ + H_2_O_(g)_ → CaO_(s)_ + 2HCl_(g)_ (2)

CaF_2(l)_ + H_2_O_(g)_ → CaO_(s)_ + 2HF_(g)_ (3)

H_2_O may be introduced into the system as a contaminant (reagents, construction materials, gases, etc.), especially since CaCl_2_ is a highly hygroscopic reagent. At process temperatures, the free enthalpy change of reactions (2) and (3) is positive, which would suggest that they should not occur. However, the gaseous products are quickly removed from the reaction zone, resulting in low partial pressures. In such cases, the reaction equilibria will shift to the right, favoring the formation of corrosive and toxic gases. These considerations were experimentally confirmed in the study [20].

The analyzed sections of the lance, L1 and L2, did not show significant wear, as they were only exposed to high temperatures and gases (mainly N_2_-CO_2_) or potentially to corrosive HCl and HF, as mentioned above. Contact with molten salts was significantly limited and could occur mainly due to gas sparging and salt splashing above the salt level. The green color of the deposit on the outer surface of the lance in sections L1 and L2 may suggest the formation of nickel chloride (NiCl_2_) as a result of the reaction (4) or nickel oxide (NiO) as a result of a reaction (5):

Ni + 2HCl_(g)_ → NiCl_2_ + H_2(g)_ (4)

Ni + 1/2O_2(g)_ → NiO_(s)_ (5)

Oxygen can be introduced into the system as a contaminant, similar to the moisture discussed above. The free enthalpy change for reaction (4) is positive under process conditions (temperature 700–940 °C) [17], whereas for reaction (5), it is negative [17]. Attempts to perform XRD analysis of the green deposit taken from zones L1 and L2 proved very challenging due to the amorphous nature of the material.

The results of the X-ray analysis presented in Figure 17 and reconstructed into a 3D model (Figure 18) revealed minor defects in the structural material (most likely originating during tube manufacturing) and defects arising during the construction of the reactor tank. The most visible of these are in the welded areas, where discontinuities in the metallic structure were detected. These are not a result of prolonged operation of the reactor in the aggressive environment of molten salts. An important finding is that no complete discontinuity occurred in any part of the reactor tank under investigation, which would risk tank leakage and reactor failure.

Using computed tomography, numerous discontinuities were revealed at the welding joints in the lower part of the reactor chamber (R3). In this component, gaps were also found at the junction of the round bottom element with the reactor tube, resulting from insufficient fitting of the components—something not visible in the X-ray images. Proper continuity of the weld was observed on the outer side of the reactor tube. Unfortunately, due to significant noise and interference in the analyzed object, it was not possible to create a three-dimensional thickness map for the R3 component. Such a map is useful, as demonstrated for components R1 and R2, as it allows the identification of wear inhomogeneity.

3D scanning provided additional information on the wear of part R3, which is the most exposed to the corrosive environment and high temperature. In section R3, negative deviations predominate on the outer side. These are mainly caused by the chipping of corrosion products (NiO) from the surface, as this side of the reactor chamber was exposed to oxidizing conditions. It is possible to prevent such strong oxidation of nickel by using an inert gas atmosphere (e.g., argon or nitrogen) injected into the space between the reactor chamber and the heating module, which should significantly limit material oxidation. On the other hand, the inner side, exposed to molten salts, also shows uneven wear. The material is subject to wear due to mechanisms described for the lance, which causes a reduction in wall thickness. However, 3D scanning takes into account the thickness of corrosion products and partially crystallized salts on the walls. Precisely determining the wear of the material itself is problematic and may not be reliable.

## 5. Conclusions

Studies of the lance operating for 800 h in the reactor chamber revealed wear on both the internal and external surfaces. This wear is primarily dependent on the direct and continuous contact of the lance with molten salts consisting of CaCl_2_-CaF_2_-CaO-CaCO_3_. Microscopic observations indicated that the upper parts of the lance (above the molten salts and at the salt/gas boundary) experienced minimal wear (109 µm/year and less).

On the external parts of the lance above the salt level, crystallized salts were observed. Their presence during continuous operation may protect the lance, resulting in low wear. For the lance in continuous contact with molten salts, wall thinning amounted to approximately 22%, with an estimated annual wear of 3.9 mm. The significant wear is attributed to the direct contact of metal with the salts and the turbulent motion caused by gas sparging.

The results of microstructure observations on the cross-sections of the lance demonstrated the presence of intergranular corrosion on both the inner and outer sides of the tubes. Corrosion was observed in the form of voids as well as a continuous network along the grain boundaries. The corrosion primarily spreads perpendicularly to the contact surface with the corrosive environment, penetrating to significant depths and locally reaching hundreds of micrometers into the material.

X-ray analysis enabled the assessment of the entire element without the need for its destruction. Discontinuities in the reactor chamber material, caused by incomplete fusion during welding, were identified. The reconstruction of X-ray images into a 3D model allowed for precise localization of these discontinuities. They were only visible on the inner sides of the reactor chamber, where they could not be welded. The external welds were found to be permanent and continuous.

Computed tomography enabled multi-plane analysis of the reactor chamber, revealing additional imperfections from the construction stage (connection of the reactor bottom with the reactor tube). Based on CT results, uneven wear of individual elements on both the inner and outer surfaces was noted. The distribution of the reactor chamber wall thickness indicates thickening in many places (presence of corrosion products) as well as thinning (pitting). Unfortunately, due to the large noise during the analysis, the result of the most complex component, R3, was difficult to interpret.

The 3D scanning served as a complementary analysis and allowed for the assessment of wear on the component most exposed to the corrosive effects of molten salts (R3). This reactor zone exhibited significant material loss on both the external and internal sides. On the external side, this was caused by the oxidizing atmosphere and the formation of nickel oxide, whose layers flaked off the reactor.

The change in the thickness of the inner part of the reactor chamber is caused by material corrosion, with a mechanism analogous to that observed in the lance. This conclusion is reached based on an analysis of part of the results (microstructural studies) and the assumption that both components (reactor and lance) exhibit the same behavior. This is justified when considering that both components were made of the same material and operated at the same temperature and in the presence of the same molten salts.

The outer part of the reactor, exposed to high temperatures and oxidizing conditions, requires inert gas protection to limit nickel oxidation. This is important for the external welds, which are a key place to ensure the reactor chamber is leak-proof.

The condition of the lance used for N_2_-CO_2_ gas sparging requires monitoring and potential replacement during long-term tests. An alternative is to use a lance with thicker walls. While this would not eliminate wear, it would reduce the frequency of replacements.

The consumption of Ni 200 Alloy found in this study is acceptable for the operation of the lance and reactor chamber. However, it seems that the wear rate found will be too high for the stable operation of more complex industrial plant components (such as molten salt pumps).

## Figures and Tables

**Table 1:** Chemical composition of Ni 200 Alloy, as provided by the vendor.

Elements	Ni	Mn	Cu	Si	C	Fe	S
wt. %	99	0.35	0.25	0.35	0.15	0.40	0.01

**Table 2:** Wall thickness measurement results on the cross-section of the examined lance before and after high-temperature tests.

Sample	Wall Thickness, µm	Wall Thinning Relative to the Original Tube, µm
L1	Average: 1645	2 (0.12%)
	Min.: 1595	
	Max.: 1692	
L2	Average: 1637	10 (0.61%)
	Min.: 1591	
	Max.: 1662	
L3	Average: 1294	353 (21.43%)
	Min.: 1212	
	Max.: 1391	
L0 (before tests)	Average: 1647	-
	Min.: 1616	
	Max.: 1678	

## Data Availability

The original contributions presented in this study are included in the article. Further inquiries can be directed to the corresponding author.
